# The knowns and unknowns of phlebotomine sand flies (Diptera: Psychodidae) in selected countries of Central Europe

**DOI:** 10.1186/s13071-025-07160-9

**Published:** 2025-11-29

**Authors:** Katharina Platzgummer, Sandra Isabell Oerther, Tomáš Bečvář, Vít Dvořák, Jovana Sádlová, Barbora Bečvářová, Petr Volf, Adelheid G. Obwaller, Karin Bakran-Lebl, Attila J. Trájer, Julia Walochnik, Edwin Kniha

**Affiliations:** 1 Institute of Specific Prophylaxis and Tropical Medicine, Center for Pathophysiology, Infectiology and Immunology, Medical University Vienna, Vienna, Austria; 2 Independent Researcher, Karlsdorf-Neuthard, Germany; 3 Department of Parasitology, Faculty of Science, Charles University Prague, Prague, Czechia; 4 Division of Science, Research and Development, Federal Ministry of Defence, Vienna, Austria; 5 Department for Vector-Borne Diseases, Austrian Agency for Health and Food Safety (AGES), Vienna, Austria; 6 Sustainability Solutions Research Lab, University of Pannonia, Veszprém, Hungary

**Keywords:** *Phlebotomus sp.*, Leishmaniasis, Rodents, Seasonality, *Trypanosoma*

## Abstract

**Background:**

Sand flies are vectors of the protozoan *Leishmania* spp. and phleboviruses. In Europe, several species are widely distributed in the Mediterranean region and a northward spread can be observed. They can be found regularly also in some regions of Central Europe, with *Phlebotomus mascittii* being the most cold-tolerant and northerly distributed species, but the knowledge on their distribution in countries such as Germany, Austria, Czechia, Slovakia, and Hungary remains fragmentary because of a lack of comprehensive field studies and a poor understanding of the ecological requirements and phylogeographic history.

**Methods:**

A comprehensive literature review of sand fly occurrence in five Central European countries was complemented by entomological surveys, including sand fly and rodent screening for sand fly-borne pathogens. Nucleic acid extraction, COI barcoding, blood meal analysis, and phylogenetic and environmental analyses incorporating unsupervised machine learning techniques were conducted.

**Results:**

This study significantly advances the understanding of the current distribution of six sand fly species in Central Europe. Among them, only *Ph. mascittii* was present in all analyzed countries, except Czechia, with its seasonal activity peaking in July. *Phlebotomus papatasi*, *Ph. perfiliewi* and *Ph. neglectus* were recorded in Hungary, while *Ph. perniciosus* and *Phlebotomus simici* were found in Germany and Austria, respectively. Although *Leishmania* DNA was absent in sand flies and rodents, DNA from two distinct *Trypanosoma* lineages was detected in several specimens, suggesting *Ph. mascittii* feeds on both birds and ruminants. Trypanosomatid lineages identified in local rodents differed, indicating distinct lineages between sand flies and rodents. Environmental analysis identified 15 Corine land cover classes associated with sand fly presence, with urban locations being the most frequently occupied. Linear regression models comparing presence versus absence revealed significantly higher sand fly presence in forested and urban landscapes. Furthermore, *Ph. mascittii* populations formed four distinct ecological clusters, which broadly grouped into two main geographic groups: one in the Upper Rhine Valley of southwestern Germany and the other spanning the Carpathian Basin.

**Conclusions:**

This study provides new insights into the current distribution, ecological preferences, seasonal activity, and potential vector capacity of sand fly species in Central Europe.

**Graphical Abstract:**

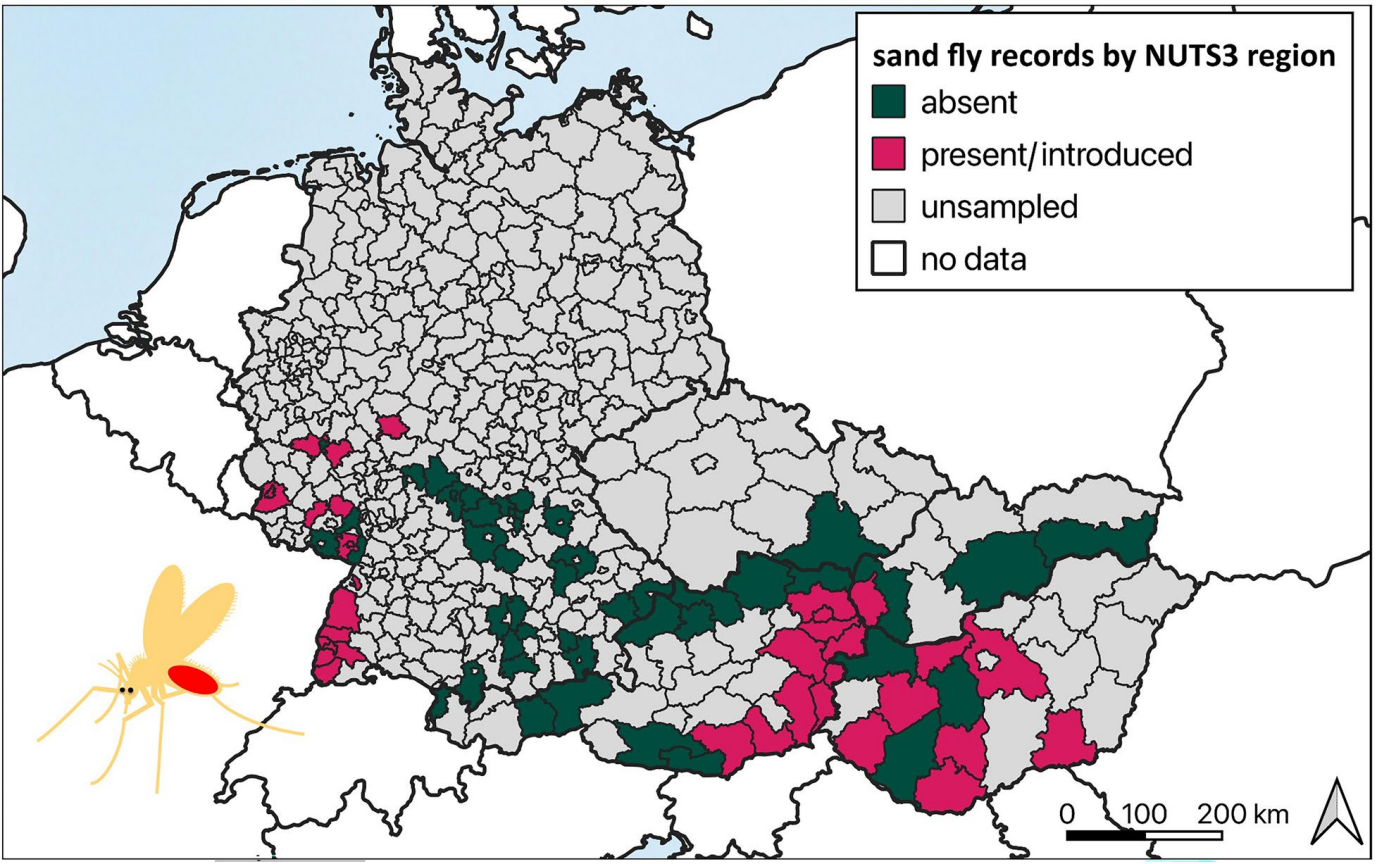

**Supplementary Information:**

The online version contains supplementary material available at 10.1186/s13071-025-07160-9.

## Background

Phlebotomine sand flies (Diptera: Psychodidae: Phlebotominae) are small, blood-feeding insects of great medical importance because they transmit the protozoan parasites *Leishmania* spp. as well as arboviruses, most importantly phleboviruses [1–3].

More than 20 *Leishmania* species potentially cause leishmaniases in humans, which are considered a neglected tropical disease (complex), with about 1 billion people being at risk of infection. In Europe, autochthonous *Leishmania* infections are common mainly in the Mediterranean area, but imported cases have also been described also in other regions, including Central Europe [3].

Besides transmitting *Leishmania* spp., sand flies are the principal vectors of phleboviruses, of which sandfly fever Sicilian virus (SFSV), Naples virus (SFNV), and Toscana phlebovirus (TOSV) are the medically most important species in Europe potentially causing 3-day fever, a mild, self-limiting febrile illness. In addition, TOSV can be neuroinvasive, causing meningitis and meningoencephalitis with increased numbers of patients in the summer months in endemic areas such as Tuscany in Italy [4–6].

Worldwide, more than 1000 species of sand flies have been described, of which 23 species of the genera *Phlebotomus* and *Sergentomyia* can be found in Europe, being highly abundant in Mediterranean countries [7], whereas the sand fly fauna of Austria, Germany, Czechia, Slovakia, and Hungary remains understudied because of only sporadic and inconsistent sampling. Of these countries, Hungary has the first records and to date the most diverse reported sand fly fauna, with *Phlebotomus perfiliewi* Parrot, 1930, *Ph. neglectus* (Tonnoir, 1921), *Ph. mascittii* Grassi, 1908, and *Ph. papatasi* (Scopoli, 1786) being endemic [8–11].

An absence of sand flies north of the Alps had been assumed until the first individuals of *Ph. mascittii* were observed in Baden-Wuerttemberg, Germany, in 1999 [12]. This marked the beginning of more intensive field studies, leading not only to the discovery of more *Ph. mascittii* populations in Germany, representing the northernmost records of sand flies in Europe, but also to the first record of *Phlebotomus perniciosus* Newstead, 1911 in Rhineland-Palatinate in 2001 and 2008 [12–17]. A decade after their first detection in Germany, sand flies were also found in Austria, first in Carinthia in 2009 and later also in all eastern federal states, where *Ph. mascittii* has then been trapped regularly [18–22]. In addition, a single female specimen of *Phlebotomus simici* Nitzulescu, 1931, was found in Orth an der Donau, Lower Austria, in 2019 [23]. In Slovakia, the detection of a female *Ph. mascittii* in the village of Pernek remains the only sand fly record in the country to date, while no sand flies have been found in Czechia yet [24]. Unlike initial assumptions of recent emergence in Central Europe associated with climate change, a recent phylogeographic study hypothesizes northward post-glacial dispersal from southern refugia and populations that have been overlooked for a long time in small climatically favored regions at the margin of their geographical distribution, regarded as non-endemic for sand fly-borne pathogens [25]. However, recent natural northward spread influenced by warming climate has been assumed in Switzerland and Italy [26, 27].

While many species of the genus *Phlebotomus* have been proven to be vectors for *Leishmania* spp. and phleboviruses, the vector competence of *Ph. mascittii*, the predominant species in Central Europe, remains unclear. The suspected vector competence is based on possible autochthonous cases of leishmaniasis in regions with *Ph. mascittii* presence, and female specimens of *Ph. mascittii* have been found positive for *Leishmania infantum* in PCR-based assays in Austria, Italy, and Slovenia [28–30].

Reliable data on the incidence of sand fly-borne diseases (SFBDs) in both humans and potential reservoir animals remain scarce in Central European countries, as surveillance programs are not in place and cases of SFBDs are not notifiable except in Czechia. Cases of autochthonous leishmaniasis were observed in a dog in Hungary [11] and dogs and horses in Germany [31]. Moreover, a *Leishmania* seroprevalence of > 20% was observed in retrospectively tested German dogs, with most positive dogs having had a history of importation or travel to endemic countries [32]. In addition, *L. infantum* seropositivity was detected in sheep from Southern Germany [33]. Autochthonous as well as imported cases of human leishmaniasis are well documented and recorded with varying frequency in Central Europe [34–39].

To date, records of sand flies in Central Europe are typically separated by long geographical distances, which raises the question of overlooked occurrences, as distribution dynamics of sand flies remain poorly understood [25]. Consequently, it is difficult to assess the risk of establishment of transmission cycles of sand fly-borne pathogens such as *Leishmania* spp. and phleboviruses. This study provides an overview and update of the distribution of sand flies in Central Europe, combining historical and recent data, including molecular screenings of sand flies, highlighting the impact of sand flies as vectors of sand fly-borne pathogens (SFBPs) on human and veterinary health and the importance of close monitoring of sand flies and SFBDs in non-endemic countries.

## Methods

### Literature search and data extraction

The literature search followed the Prisma Journal Publishing protocol workflow [40] and focused on five Central European countries, namely Austria, Czechia, Germany, Hungary, and Slovakia, as studies focusing on other Central European countries (Switzerland, Luxembourg, and Belgium) have been published recently [26, 41, 42]. PubMed, Web of Science, Ovid Medline, CAB Direct, and Google Scholar databases and web searches were screened up to 2025. Full text articles, reports, theses, congress presentations, and book chapters in English, German, Slovak, Czech, and Hungarian languages containing information on phlebotomine sand flies from Austria, Czechia, Germany, Hungary, and Slovakia were selected. Some data originated from direct consultations with experts and their in-house unpublished databases. The following search string was used. Terms in title: ("Phlebotominae" OR "sand flies" OR “sandflies” OR "Phlebotomus") AND ("Austria" OR "Germany" OR "Czech Republic" OR "Slovakia" OR "Hungary" OR "Central Europe").

### Sand fly surveys

For this study, data from five surveys were combined (Table 1).

**Table 1:** Trapping information of all sand fly surveys included in the study

Survey	Country	Type of trapping	Time	Number locations	Number trap nights	Number sand flies	Sand flies/trap night
#1	Austria	Regular	2018–2019	4	801	450	0.56
#2	Austria	Sporadic	2020	3	68	136	0.50
#3	Austria	Regular	April–Oct. 2023	8	204	31	0.15
			May–Oct. 2024	8	188	42	0.22
		Sporadic	July 2023	17	37	1	0.03
			June–Aug. 2024	55	198	2	0.01
#4	Germany	Regular	May–Sept. 2023	12	214	42	0.20
			May–Sept. 2024	10	174	39	0.22
		Sporadic	Aug.–Sept. 2023	1	4	0	0.00
			July 2024	6	23	27	1.17
#5	Czechia	Regular	May–Sept. 2023, 2024	3	162	0	0
		Sporadic	July–Aug. 2024	5	21	0	0

Survey #1: 450 *Ph. mascittii* caught in 2018 and 2019 in Styria and Lower Austria were screened for Trypanosomatidae DNA [22].

Survey #2: 136 *Ph. mascittii* were trapped during sporadic trappings in 2020 at three previously reported locations in Styria and screened for Trypanosomatidae DNA (unpublished).

**Fig. 1:**
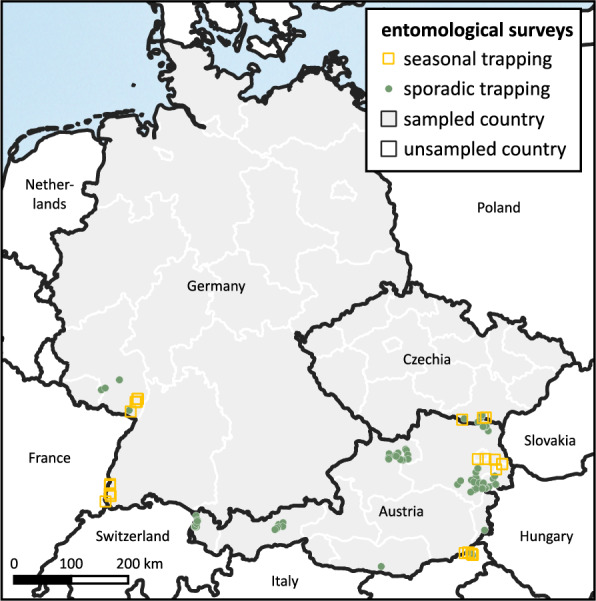
Trapping sites of sand fly surveys presented in the study. Seasonal trappings were conducted over several months in two trapping years, 2023 and 2024, in Austria, Czechia, and Germany. Sporadic trappings were performed for 1 or 2 nights in Austria and Germany

Surveys #3 to #5: Recent entomological surveys were conducted in 2023 and 2024 in the frame of the CLIMOS project (https://climos-project.eu/) at locations in Austria (survey #3), Germany (survey #4), and Czechia (survey #5). Thereby, two trapping approaches were followed: (i) Regular trappings were conducted monthly between April and October for 2 consecutive days at 8 previously sampled locations in Austria, 12 locations in Germany, and 3 locations in Czechia. (ii) Additionally, sporadic trappings to find new locations were conducted in July and August for one or two nights at rural and peri-urban locations (Fig. 1). Mainly private properties with animals and animal farms were chosen for trapping. Informed consent was obtained from all landowners. Variables such as coordinates, locality type, trap position, and animal presence were always registered (Supplementary Table 1).

For all surveys, one to eight CDC miniature light traps (John W. Hock Co., Gainesville, FL, USA) were placed at each location depending on the size of the locality and numbers of buildings and potential reservoir hosts (Supplementary Table 1).

### Rodent sampling

Rodents were sampled with live traps at two locations in Styria (Unterpurkla and Hummersdorf) from 16–19 September 2020. Both locations had exhibited the highest sand fly abundance in previous surveys, and rodent presence was universally recorded at all sites and surveys and might thus be most suitable for potential pathogen transmission [21, 22]. Trapped specimens were killed by cervical dislocation under anesthesia (mixture of 62.5 mg/kg ketamine and 25 mg/kg xylazine). Species identification was based on morphological criteria using the key by Anděra and Horáček [43]. All individuals were weighed, and their body and tail lengths, ear length, and hind paw length were measured. Ear pinnae, ear-draining lymph nodes, spleen, liver, paws, and tail skin were collected and immediately stored dry at − 20 °C for molecular analyses.

### Sand fly dissection and identification

Sand flies were dissected, and heads and terminal abdominal segments were slide-mounted in CMCP-10 mountant (Polysciences, Inc., Warrington, PA, USA). Identification was based on descriptions of male genitalia, female spermatheca, and pharyngeal armature in published morphological keys [44, 45].

Remaining sand fly body parts were stored individually in 2.0-ml tubes and stored at − 80 °C for nucleic acid extraction.

### Nucleic acid extraction

All specimens were homogenized individually in 500 µl Dulbecco’s modified Eagle medium (DMEM) supplemented with 20% bovine serum albumin, 10 µg/ml gentamicin, 0.25 µg/ml amphotericin B, and 1% penicillin/streptomycin (all from Gibco, Thermo Fischer Scientific). Two metal beads (3 mm diameter) were added to each tube and beat with a TissueLyser bead mill (QIAGEN GmbH, Hilden, Germany), shaking at 50 Hz for 2 min (min). The homogenate was cleared by centrifugation for 5 min at 8000 rpm at 4 °C in a tabletop centrifuge.

Two hundred microliters of each homogenate was used for nucleic acid extraction. For sand flies caught in Styria in July 2024, homogenates from up to five individuals were pooled by species and sex with a total volume of 200 µl of homogenate per extraction. Nucleic acids were extracted using a QIAmp^®^ RNeasy mini kit 250 (Qiagen, Hilden, Germany) with a final elution volume of 50 µl following the manufacturer’s protocol with adjustments based on previous tests. Based on a recently performed External Quality Assessment [46], the QIAmp® RNeasy mini kit without using DNase is sufficient for DNA as well as RNA-based screenings. DNA and RNA yield and quality were checked with a NanoDrop ND-1000 (Thermo Fisher Scientific Inc., Waltham, MA, USA).

### DNA-based pathogen screening

Individual and pooled females were screened for *Leishmania* spp. in a probe-based qPCR protocol targeting the kinetoplast DNA (kDNA) using primers and probe from Mary et al. [47], using a Luna® Universal Probe qPCR Master Mix (New England Biolabs, Ipswich, MA, USA) with a final volume of 20 µl and a Bio-Rad CFX96 Touch Real-Time PCR Detection System (Bio-Rad Laboratories, Inc., Hercules, CA, USA) with an initial cycle of 95 °C for 3 min followed by 45 cycles each consisting of 95 °C for 15 s and 60 °C for 1 min. In addition, a nested PCR targeting the 18S rRNA gene was applied to detect Trypanosomatidae DNA, using the PCR conditions and primer combinations Tryp_18S_F1/Tryp_18S_R1 and Tryp_18S_F2 /Tryp_18S_R2 as described by Peña-Espinoza et al. [48]. In all reactions, negative (sterile H_2_O) and positive controls (*L. infantum* DNA corresponding to 10.2 parasites/ml) were used.

All conventional PCR products were analyzed using a Gel Doc™XR + Imager (Bio-Rad Laboratories Inc., Hercules, CA, USA), and amplified fragments were purified using an Illustra™ GFX™ PCR DNA and Gel Purification Kit (GE Healthcare, Buckinghamshire, UK). The samples were sent to Microsynth (Microsynth Austria GmbH, Vienna, Austria) for Sanger sequencing, and the obtained bidirectional sequences were aligned using ClustalX 2.1 [49]. Consensus sequences were generated using GeneDoc 2.7.0 [50]. Thereafter, sequences were blasted and compared to minimum and maximum top 100 hits with > 86% query coverage in the NCBI sequence database.

### Molecular typing of Trypanosomatidae

Available sequences for comparison were downloaded from GenBank and aligned with the obtained sequences using ClustalX 2.1 for multiple alignments and GeneDoc 2.7.0 for manual editing and data analysis. DnaSP v.5 [51] was used to identify unique haplotypes. A neighbor-joining (NJ) tree was calculated in MEGA X [52] using uncorrected pairwise distances with bootstrap support of 1000 replications to illustrate genetic relatedness between analyzed Trypanosomatidae sequences.

### RNA-based pathogen screening

All individuals and pools were screened for phlebovirus RNA using three different protocols. A one-step RT-PCR protocol targeting various phleboviruses was used following the protocol of Matsuno et al. [53], applied using a SuperScript™ One-Step RT-PCR System with Platinum^™^ Taq DNA polymerase (Thermo Fischer Scientific) with a final volume of 25 µl. Additionally, two probe-based RT-qPCR protocols were applied to screen for Toscana virus (TOSV, *Phlebovirus toscanaense*) [54–56] and sandfly fever Sicilian virus (SFSV; *Phlebovirus siciliaense*) [57] separately with the primer sets and concentrations given in Ayhan et al. [58] using a Luna® Universal Probe One-Step RT-qPCR Kit (New England Biolabs, Ipswich, MA, USA) and a Bio-Rad CFX96 Touch Real-Time PCR Detection System (Bio-Rad Laboratories, Inc., Hercules, CA, USA) with one cycle at 55 °C for 10 min and a cycle at 95 °C for 1 min followed by 45 cycles each consisting of 95 °C for 10 s and 60 °C for 45 s. Negative (sterile H_2_O) and positive controls (for Pan-Phlebovirus RT-PCR: Punique virus (PUNV Tunisie2009 T101 strain), for TOSV RT-qPCR: Toscana virus (MRS2010 Marseille 2010 strain), for SFSV RT-qPCR: Sandfly fever Sicilian virus (Italy 1943 Sabin strain) were used for all assays.

### COI barcoding and blood meal analysis

In case of unsuccessful morphological species identification, a barcoding PCR was performed targeting a 658-base pair (bp) fragment of the cytochrome c oxidase subunit I (COI) gene with the primers LCO1490 (5’-GGTCAACAAATCATAAAGATATTGG-3’) and HCO2198 (5’- TAAACTTCAGGGTGACCAAAAAATCA-3’) published by Folmer et al. [59], with the following PCR conditions: 94 °C for 5 min, 35 cycles at 94 °C for 1 min, 52 °C for 1 min and 72 °C for 1 min, followed by a final elongation of 72 °C for 10 min. For blood meal analysis, two PCR protocols were applied. First, a PCR targeting a 16S rDNA fragment with the primers L2513 (5’-GCCTGTTTACCAAAAACATCAC-3’) and H2714 (5’-CTCCATAGGGTCTTCTCGTCTT-3’) published by Kitano et al. [60], with the following PCR conditions: 94 °C for 5 min, 35 cycles at 94 °C for 30 s, 57 °C for 15 s, and 72 °C for 30 s, followed by a final elongation at 72 °C for 10 min. Additionally, an avian-specific PCR targeting a 220-bp fragment of the cytochrome b (Cytb) gene using the primer combination L15330AV (5’-GGACAAATATCATTCTGAGG-3’) and H15551AV (5’-GGGTGGAATGGGATTTTGTC-3’) published by Lee et al. [61] was applied, with the following PCR conditions: 94 °C for 5 min, 35 cycles at 94 °C for 30 s, 60 °C for 45 s, and 72 °C for 1 min, followed by a final elongation of 72 °C for 7 min. Barcoding and bloodmeal PCRs were performed using a 2 × EmeraldAmp® GT PCR Master Mix (optimized buffer, PCR enzyme, dNTP mixture, green gel loading dye) (Takara Bio Europe AB, Göteborg, Sweden) with 5 µl template DNA and a final volume of 25 µl and an Eppendorf Mastercycler (Eppendorf AG, Hamburg, Germany). Bands were cut out from the gel, purified, and sent for Sanger sequencing as stated above (DNA-based pathogen screening).

### Environmental analyses

To analyze environmental factors associated with presence/absence of sand flies, we accessed CORINE land cover (CLC) data from 2018 (https://land.copernicus.eu/en/products/corine-land-cover), presented as 44 different categories. To determine the degree of urbanization at positive and negative trapping sites, we accessed Global Human Settlement Layer data (GHS-SMOD) (https://human-settlement.emergency.copernicus.eu) from Pesaresi et al. [62]. Based on eight class descriptions, we determined simplified categories: urban, peri-urban, rural, and uninhabited (Table 2). Elevation data were obtained from the ETOPO Global Relief Model [63]. Rainfall erosivity data were sourced from the European Soil Data Centre (ESDAC) of the European Commission, based on the work of Panagos et al. [64]. To extract data for all trapping sites, raster values were sampled with QGIS 3.34.1.

**Table 2:** Global Human Settlement Layer data (GHS-SMOD) categories

Code	Class description	Simplified category
30	Urban center	Urban
23	Dense urban cluster	Urban
22	Semi-dense urban cluster	Urban
21	Suburban or peri-urban	Peri-urban
13	Rural cluster	Rural
12	Low density rural	Rural
11	Very low density rural	Rural
10	No population (uninhabited)	Uninhabited

### Climate classification and bioclimatic variables

Climate zones within the study area were categorized using the Köppen-Geiger classification map at 1-km resolution, developed by Beck et al. [65], based on the WorldClim version 2.1 dataset [66]. This classification scheme provides a categorical representation of prevailing climatic conditions.

To characterize the climatic conditions associated with sand fly occurrence, 19 standard bioclimatic variables were extracted from WorldClim version 2.1 [66]. These variables represent long-term averages for the period 1970–2000 and include key metrics related to temperature and precipitation seasonality and extremes. The grid-based map for the biogeographical regions of Europe was used to characterize the biome-based occurrence patterns of sand flies [67].

### K-means clustering

K-means clustering was applied to group occurrence sites into distinct environmental clusters [68]. The algorithm iteratively assigned data points to the nearest centroid across *k* clusters, minimizing intra-cluster variation. The optimal *k* was determined using the elbow method (based on within-cluster sum of squares) and silhouette score, which respectively measure compactness and separation [69]. Clustering results were visualized to identify underlying ecological groupings.

### Principal coordinate analysis (PCoA)

Principal coordinate analysis (PCoA) [70] was used to explore similarities related to sand fly occurrence sites based on three types of numerical (elevation, rainfall erosion index, and 19 bioclimatic) and three types of categorical (FAO soil orders, Copernicus CLC, and Köppen-Geiger climatic classes) variables. Categorical variables were encoded using the one-hot encoding process. This ordination method projects multivariate dissimilarities into a reduced-dimensional space, facilitating pattern recognition. A distance matrix—typically using Bray-Curtis for ecological data or Euclidean for continuous variables—was computed and used for classical (metric) PCoA via eigenvalue decomposition [71]. The first two axes, capturing the highest variance, were visualized to reveal spatial patterns such as clustering or gradients.

### Decision tree analysis

A decision tree classifier [72] was used to interpret the k-means clustering results. Environmental and categorical variables (e.g. climate, land cover, soil) were pre-processed, and clusters were used as target classes. The tree revealed which variables most effectively distinguished the clusters, providing an interpretable model of cluster membership. This approach bridges unsupervised structure detection with supervised rule-based interpretation, offering insights into the environmental drivers of leishmaniasis distribution.

### Mapping of sand fly records

Sand fly records from previous and recent surveys were incorporated into distribution maps. Therefore, exact coordinates were extracted and converted to the NUTS3 level (https://gisco-services.ec.europa.eu/distribution/v1/nuts-2024.html) and classified based on an updated scheme used for the phlebotomine sand fly maps of the European Center for Disease Prevention and Control (ECDC) (https://www.ecdc.europa.eu/en/disease-vectors/surveillance-and-disease-data/phlebotomine-maps) (Table 3).

**Table 3:** Classifications of mapped sand fly records at NUTS3 level

Status	Description by ECDC	Updated description
Introduced	The species has been introduced in the administrative unit without confirmed establishment	The species has been introduced (one record) in the administrative unit without confirmed establishment. Follow-up surveys required!
Confirmed present	Not available	The species has been observed to be present in ≥ 2 years during entomological surveys in at least one municipality within the administrative unit
Observed absent	The species has never been reported within the administrative unit, and there have been field surveys or studies on sand flies within the last 5 years of the distribution status date	The species has never been reported within the administrative unit, and there have been field surveys or studies on sand flies within the last 10 years of the distribution status date
Follow-up absent	Not available	The species has been introduced (one record) in the administrative unit, with follow-up surveys showing absence
No data	No sampling has been performed, and no data on the species are available	No sampling with CDC miniature light traps (model #512) has been performed, and no data on the species are available

## Results

### New sand fly records and updated distribution

During entomological surveys #3 to #5, sporadic trappings at 79 new locations were performed (5 in Czechia, 3 in Germany, and 71 in Austria), of which 4 (5.1%) were positive for sand flies, namely three locations in Austria and one in Germany. At each location, one female specimen identified as *Ph. mascittii* was trapped.

**Fig. 2:**
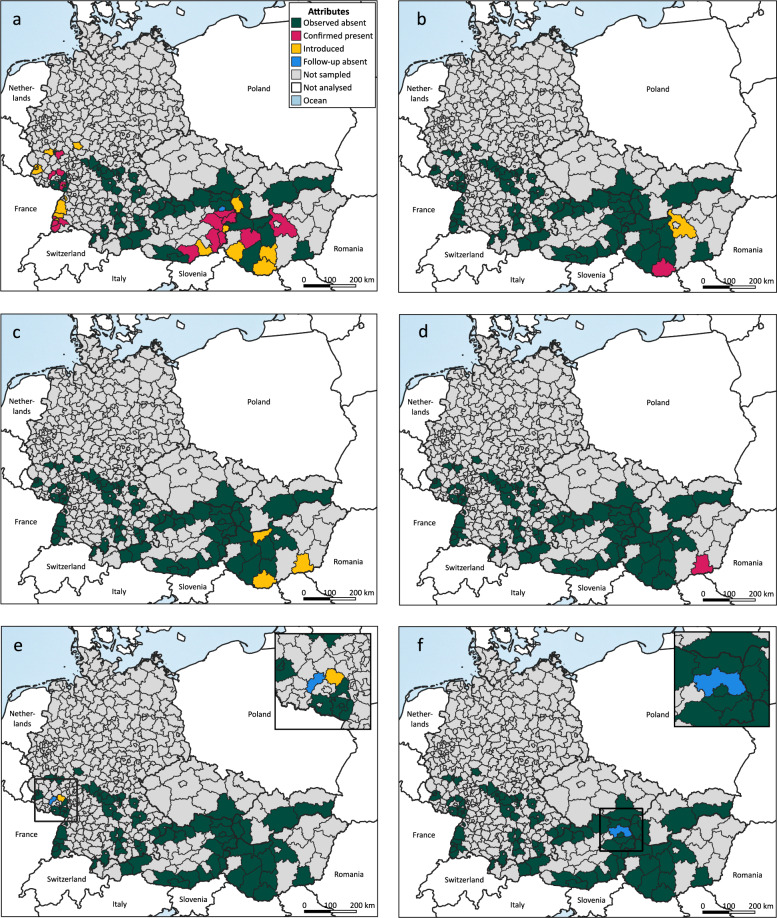
Current reported distribution of six sand fly species by NUTS3 region in five countries, Austria, Czechia, Germany, Hungary, and Slovakia, based on the adapted classification scheme used by ECDC. *Phlebotomus mascittii* (**a**), *Ph. neglectus* (**b**), *Ph. papatasi* (**c**), *Ph. perfiliewi* (**d**), *Ph. perniciosus* (**e**), and *Ph. simici* (**f**). Observed absent: The species has never been reported within the administrative unit, and there have been field surveys or studies on sand flies within the last 10 years of the distribution status date. Confirmed present: The species has been observed to be present in ≥ 2 years during entomological surveys in at least one municipality within the administrative unit. Introduced: The species has been introduced (one record) in the administrative unit without confirmed establishment. Follow-up surveys required. Follow-up absent: The species has been introduced (one record) in the administrative unit, with follow-up surveys showing absence

Overall, we retrieved 685 records, of which 116 were presence (77 introduced and 39 confirmed present) and 569 were absence records (562 observed absent and 7 follow-up absent in 80 NUTS3 regions) from 23 publications (including this study). Based on our classification system, *Ph. mascittii* showed the most introduced (57) and confirmed present (29) as well as follow-up absent (5) records (Table 4). Only *Ph. mascittii*, *Ph. neglectus*, and *Ph. perfiliewi* were classified as confirmed present, whereas *Ph. mascittii*, *Ph. perniciosus*, and *Ph. simici* had records of follow-up absence, with *Ph. simici* marking a single record in the five reported countries (Table 4, Fig. 2).

**Table 4:** Sand fly presence and absence on NUTS3 level by species

Species	Observed absent (NUTS3)	Introduced (NUTS3)	Confirmed present (NUTS3)	Follow-up absent (NUTS3)
*Phlebotomus mascittii*	594 (49)	57 (15)	29 (14)	5 (1)
*Ph. neglectus*	571 (77)	9 (1)	5 (1)	–
*Ph. papatasi*	680 (76)	5 (3)	–	–
*Ph. perfiliewi*	576 (77)	4 (1)	5 (1)	–
*Ph. perniciosus*	682 (77)	2 (1)	–	1 (1)
*Ph. simici*	684 (78)	–	–	1 (1)

### Sand fly activity

**Fig. 3:**
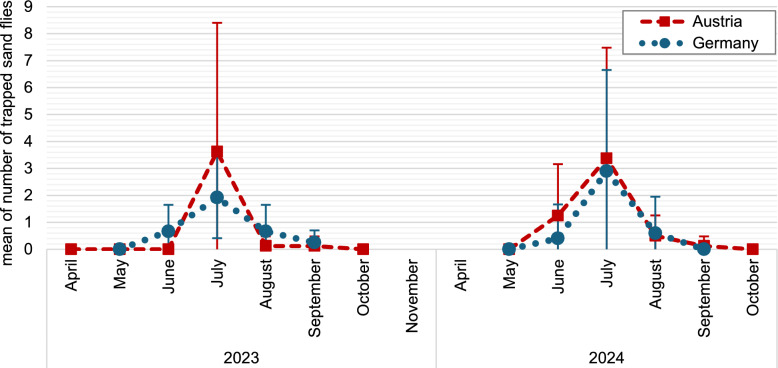
Mean and standard deviation of number of sand fly individuals trapped across all locations in Austria (*N* = 8) and Germany (*N* = 12) during monthly trappings in 2023 and 2024

Of 23 regularly monitored locations, sand flies were caught at 15 (65%). No sand flies were caught at the Czech trapping sites. In Austria and Germany, sand fly activity was recorded between June and September in both years, with peak activities in July at all positive locations. No sand fly activity was observed in May or October (Fig. 3).

### Environmental analysis

Due to the low sample size of sand fly species other than *Ph. mascittii*, environmental analyses are based on all six species. Altogether trapping efforts in 15 CLC categories were identified, presence records (including follow-up absence) were found in 12 (80.0%) categories, while absence data were observed in 14 (93.3%). For both presence and absence records, the two CLC categories with the highest trapping efforts were “discontinuous urban fabric” (87/123, 70.7% presence; 278/562, 49.5% absence) and “non-irrigated arable land” (12/123, 9.8% presence; 110/562, 19.6% absence).

For statistical comparison by linear regression of presence and absence data of all six species, combined CLC categories were analyzed. Of these, the categories “urban” (88/123, 71.5% presence; 288/562, 51.2%) and “agriculture” (23/123, 18.7% presence; 226/562, 40.2%) were the most sampled (Table 5). In our linear regression model to compare presence and absence, we used “agricultural land” as a reference variable. In comparison, significantly higher sand fly presence compared to absence was observed in the categories “forest” (OR = 3.1, *P* = 0.028) and “urban” (OR = 3.0, *P* < 0.001), while “semi-natural vegetation” was not significant; a single absence record in the category “wetlands” did not allow statistical inference (Table 5, Supplementary Table 2). No significant difference in sand fly presence was observed between combined population density factors “urban,” “peri-urban,” and “rural” (Supplementary Table 3).

**Table 5:** Trapping records and logistic regression parameters by combined CLC category

CLC combined	Presence (%)	Absence (%)	Total (%)	Odds ratio (95% CI)	*p*-value
Agricultural land	23 (18.7%)	226 (40.2%)	249 (36.4%)	Reference	
Forest	6 (4.9%)	19 (3.4%)	25 (3.6%)	3.10 (1.13–8.49)	0.028
Semi-natural vegetation	6 (4.9%)	28 (5.0%)	34 (5.0%)	2.11 (0.79–5.78)	0.137
Urban	88 (71.5%)	288 (51.2%)	376 (54.9%)	3.00 (1.86–4.82)	< 0.001
Wetlands	–	1 (0.2%)	1 (0.1%)	0.00001 (na)	0.983
Total	123 (100%)	562 (100%)	685 (100%)		

### K-means clustering results

**Fig. 4:**
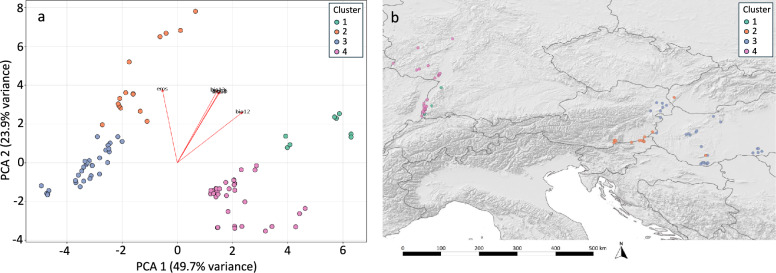
Ecological clusters of sand flies determined by k-means clustering (**a**) and clusters projected to a map of Central Europe (**b**). Factors on the biplot vectors: eros = precipitation erosivity, bio12 = annual precipitation, bio13 = precipitation of wettest month, bio16 = precipitation of wettest quarter, bio18 = precipitation of warmest quarter

Both the elbow method and silhouette scoring indicated that the optimal number of ecological clusters of sand flies in Central Europe is 4, where Clusters 2 and 3 as well as Clusters 1 and 4 formed two close ecological groups (Supplementary Fig. 1, Fig. 4).

Determined ecological clusters appeared in two distinct regions, one in the southwestern part of Germany, concentrating in and around the Rhine Valley, including Baden-Wuerttemberg and Rhineland-Palatinate, and the Saarland states of Germany. Another main ecological group is located in the Carpathian Basin, including Lower Austria and Burgenland, Transdanubia, in Austria and Hungary. Styria in Southeast Austria formed a distinct geographical subgroup (Fig. 4).

Sites in Clusters 1 and 2 are located entirely within the Continental biogeographical region of Europe. The sites in Lower Austria, Burgenland, and eastern Styria are also located within this zone. Among sites in clusters 3 and 4, all Hungarian occurrences are situated in the Pannonian biogeographical region. In contrast, sites with sand fly presence in Slovakia and Carinthia, Austria, are located in the Alpine biogeographical region (Fig. 4, Supplementary Fig. 2).

### Decision tree results

**Fig. 5:**
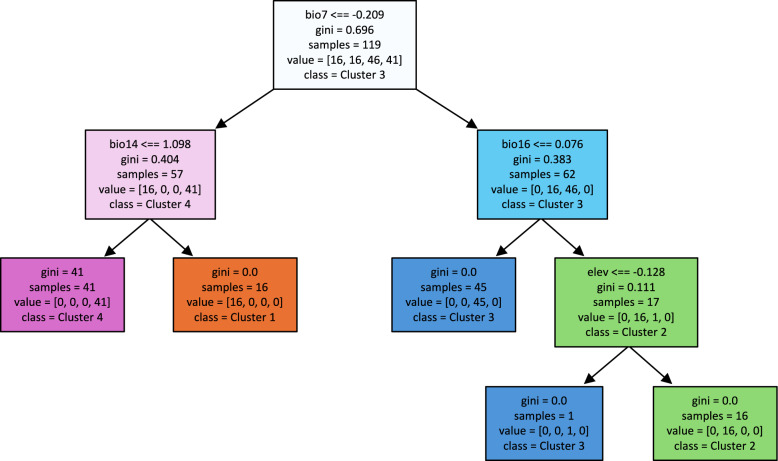
The results of decision tree analyses related to the ecological conditions of analyzed sand fly presence. Factors: bio7 = temperature annual range, bio14 = precipitation of driest month, bio16 = precipitation of wettest quarter, elev = elevation above sea level

The dominant factor discriminating between clusters was the annual range temperature (bio7). Precipitation of the driest months split ecological cluster 1 from cluster 4. Precipitation of the wettest quarter and elevation in the lower decision level discriminated between cluster 2 and cluster 3 (Fig. 5).

### Blood meals

A total of four engorged *Ph. mascittii* specimens with varying degrees of blood digestion were caught during the CLIMOS monitoring: one in Vögisheim (Germany) in August 2023, one each in Bremgarten and Hartheim (Germany) in July 2024, and one in Hummersdorf (Austria) in July 2024. Amplification of the host DNA was only successful for the Austrian specimen, namely cattle (*Bos taurus*), occurring in the vicinity of the trapping site.

### Pathogen screening

Of all 573 screened female *Ph. mascittii*, 7 (1.2%) were positive for Trypanosomatidae DNA, exclusively trapped in surveys #1 and #2 at Rohrau, Ratzenau, Hummersdorf, and Laafeld (Table 6). Neither *Leishmania* DNA nor Phlebovirus RNA was detected.

**Table 6:** Detected Trypanosomatidae DNA in female *Phlebotomus mascittii* from surveys #1 and #2

Accession	Location	Pathogen	Identity	Reference
PV560246	Rohrau	*Trypanosoma theileri* complex	99.89%	OM256572, OM256677
PV560247	Rohrau	*Trypanosoma culicavium*	99.89%	HQ107970, OM509727
PV560248	Ratzenau	*Trypanosoma theileri* complex	99.89%	OM256572, OM256677
PV560249	Hummersdorf	*Trypanosoma theileri* complex	99.66%	OM256572, OM256677
PV560250	Hummersdorf	*Trypanosoma theileri* complex	100%	OM256572, OM256677
PV560251	Hummersdorf	*Trypanosoma theileri* complex	99.78%	OM256572, OM256677
PV560252	Laafeld	Trypanosomatidae sp.	98.51%	MT241902

All together, 87 small mammals were trapped over 3 nights at two locations, comprising 27 *Apodemus flavicollis*, 14 *Clethrionomys glareolus*, 45 *Mus musculus*, and 1 *Sorex araneus* (Table 7). All except three juvenile *A. falvicollis* specimens were adults. In 5 of 87 (6.9%) specimens, Trypanosomatidae DNA was detected. Only *A. flavicollis* (4/27, 14.8%) and *C. glareolus* 2/14, 14.3%) trapped in Hummersdorf were positive (Table 7). In one *A. flavicollis* specimen (AUT093), a co-infection with two species was detected, namely *Blastocritidia* sp. in ears and paws and *Trypanosoma thomasbancrofti* in the liver (Table 7).

**Table 7:** Trapped rodent species and identity of detected Trypanosomatidae DNA

Accession	Rodent species	Organ	Pathogen	Identity	Reference
PV560253	*Apodemus flavicollis*	Ear	*Blastocrithidia* sp.	100%	GU059558, JQ658850
PV560254		Liver	*Trypanosoma thomasbancrofti*	99.89%	KT728381, KT728394
PV560255		Paw	*Blastocrithidia* sp.	100%	GU059558, JQ658850
PV560256	*A. flavicollis*	Liver	*T. thomasbancrofti*	99.89%	KT728381, KT728394
PV560257		Spleen	*T. thomasbancrofti*	99.89%	KT728381, KT728394
PV560258	*Clethrionomys glareolus*	Spleen	*Blastocrithidia* sp.	98.72%	GU059558, JQ658850
PV560259	*A. flavicollis*	Spleen	*Blastocrithidia* sp.	98.72%	GU059558, JQ658850
PV560260	*C. glareolus*	Paw	*Trypanosoma* sp.	99.67%	MH424250, MH424224

### Molecular typing of Trypanosomatidae

**Fig. 6:**
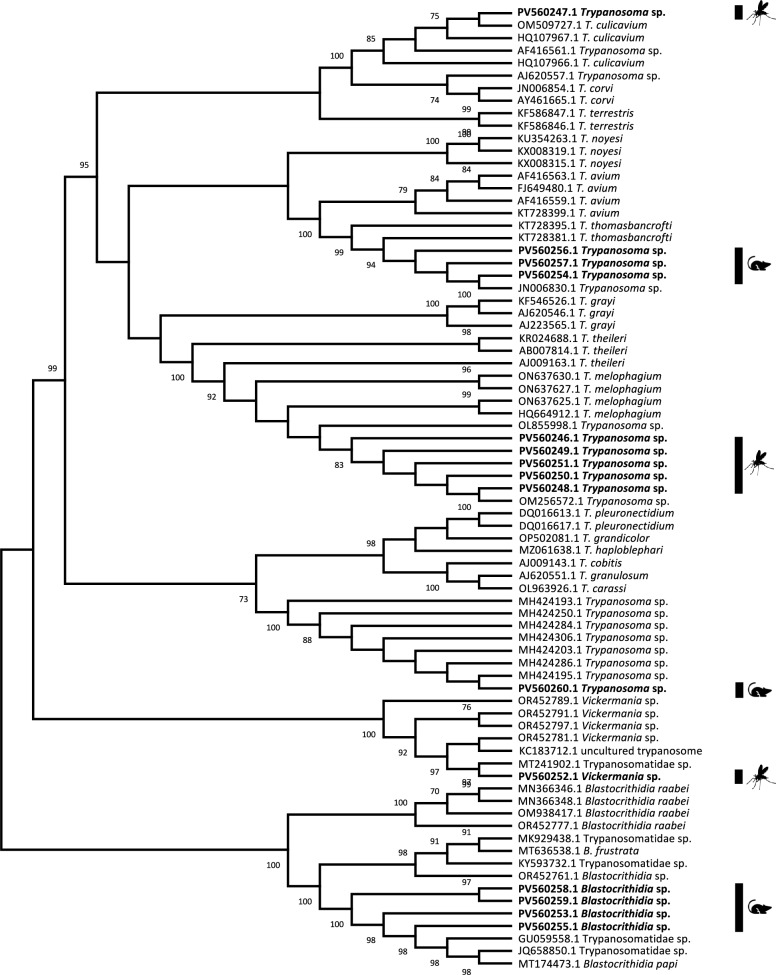
Neighbor-joining (NJ) tree including all Trypanosomatidae DNA-positive sand flies and rodents (highlighted in bold) based on 18S rDNA sequences. Symbols indicate sequences originating from either sand fly vector or rodent host. Bootstrap values > 70% are indicated next to the branches

All 15 generated Trypanosomatidae sequences were compared to 76 Trypanosomatidae sequences by creating a neighbor-joining tree using uncorrected *p*-distances. Sequences originating from sand flies and rodents were present in various major clades of the tree but were not closely related to each other (Fig. 6). Sand fly-borne trypanosomatid sequences were observed in three major clades. One clade including a single sand fly-borne sequence comprised bird trypanosomes *Trypansoma culicavium and T. corvi*. Another major clade, comprising sequences of the *Trypanosoma theileri* complex, included five sand fly-borne *Trypanosoma* sequences. One sand fly-borne sequence belonged to a clade comprising *Vickermania* sequences (Fig. 6).

Similarly, rodent-borne Trypanosomatidae sequences were observed to belong to three major clades. Three rodent-borne *Trypanosoma* sequences were most closely related to *T. thomasbancrofti*, one sequence was found to belong to a clade comprising mostly fish-borne trypanosomes (e.g. *T. carassi*, *T. grandicolor*), and four sequences belonged to a clade comprising *Blastocrithidia* (Fig. 6).

## Discussion

While phlebotomine sand flies are generally regarded as a typical element of the Mediterranean insect fauna in Europe, ongoing climatic and environmental changes strongly foster scenarios in which they become established further north, well off their traditional geographical range. Being vectors of several pathogens, they may challenge both human and animal health in countries with lacking or low awareness of their importance. In this study, we provide an update on the distribution of sand flies and SFBDs in Central Europe, i.e. Austria, Czechia, Germany, Hungary, and Slovakia.

To date, no sand flies have been detected in Czechia despite several attempts at environmentally and climatically suitable sites, and only a single female *Ph. mascittii* specimen has been collected in Slovakia, in 2016, near the Austrian border [24]. Nonetheless, stable *Ph. mascittii* populations have been reported from Austria, Germany, and Hungary. Among these, Hungary has the highest diversity of four sand fly species, with *Ph. mascittii* being widespread across several regions, while *Ph. neglectus*, *Ph. perfiliewi*, and *Ph. papatasi* have only been detected in two and three NUTS3 regions, respectively. For *Ph. neglectus* and *Ph. papatasi*, the latter of which has one of its northernmost occurrences in Hungary, there are large geographical distances between regions with recorded presence, highlighting potential underreporting across the country [73]. In contrast, sand fly distribution in Austria and Germany is localized to climatically favorable regions in southwestern Germany and (south-)eastern Austria. Only a single female specimen of *Ph. simici* was trapped in Austria [23] and has not been recorded again despite follow-up surveys at the site. There are a few records of *Ph. perniciosus* from Germany, but they have not been confirmed by later trapping efforts [15, 74].

*Phlebotomus mascittii* is the only species predominant across Central Europe [25], making it suitable for cross-border analysis. These distribution patterns align with monitoring efforts that typically focus on biogeographic regions with previously confirmed sand fly presence or ecological conditions known to be suitable for sand flies. Field work conducted in 2024 in five Austrian federal states supports this approach by showing that previously unknown sand fly locations were only found in these regions, while observed absence was confirmed in biogeographic regions influenced more strongly by the Alps, including locations in Upper Austria, Tyrol, and Vorarlberg – even though they do meet the basic requirements such as minimum temperature [21]. This results in large geographical gaps in the known distribution area across Central Europe, which can be partially explained by post-glacial repopulation of suitable habitats but could also result from a sampling bias [25].

Although *Ph. mascittii* has been trapped regularly in Germany and Austria, this species occurs in lower abundances compared to other sand fly species in Mediterranean countries. However, even in highly endemic countries, this species usually appears in numbers lower than others among the overall catch [75]. Possible explanations, apart from less suitable climatic conditions in Central Europe, could be that *Ph. mascittii* generally forms smaller populations than other sand fly species, which would also explain why they appear to be less competitive when other species are present [45, 76]. The species may differ in its habitat preferences as suggested by the cavernicolous occurrence of other species within the subgenus *Transphlebotomus* [77, 78]. Finally, *Ph. mascittii* may be less attracted to light and/or CO_2_ compared to other sand flies, and thus traditional trapping methods may underestimate its population density [79, 80]. However, all these are currently only hypotheses yet to be confirmed. In contrast, the sporadic trappings of *Ph. perniciosus* in Germany [15, 74] and *Ph. simici* in Austria [23] could not be reconfirmed when resampling the same locations in 2023 and 2024. This illustrates a fundamental problem in sand fly monitoring, namely the lack of a universal system for categorization of presence/absence data that includes the possibility that species can retreat again from sites that were previously confirmed positive. Due to the unclear mode of dispersal and different ecology compared to other vectors such as mosquitoes, including habitat requirements and breeding site preferences, the classification used by the ECDC into “Introduced,” “Observed absent,” and “No data” might be insufficient for sand flies. We therefore stress the need to perform follow-up surveys at “Introduced” sites to confirm established populations and potential transmission cycles. In Germany and Austria in particular, several locations where sand flies had been trapped in the past were found to be negative after some years. Apart from the possibility that individual sand flies may be overlooked, especially when population density is low, the absence of sand flies from previously positive locations during follow-up surveys could be attributed to only temporary occupation by sand flies, which were unable to reproduce and establish a permanent population because of unsuitable ecological conditions or changes in the ecological suitability. It is also possible that natural drift of habitat requirements of filial generations differ from the parental generation; this could lead to natural habitat drift or dispersal. Another explanation is recent anthropogenic import of a few or even a single sand fly, for example as larvae in soil [25, 81]. However, these factors are still poorly understood because of limited knowledge on breeding site preferences. To better assess these, the life cycle of sand flies in the field must be further investigated, which should also include the development of alternative sampling methods for sand flies such as the screening for larvae in soil samples using molecular techniques.

The importance of microclimatic differences is corroborated by our observation that annual range temperature and precipitation are the main variables discriminating the clusters of sand flies that can be observed in Central Europe. For a better understanding of the interrelation of the spread of sand flies and the dispersal of SFBPs, further investigations into the environmental preferences of sand flies are crucial. To enable more detailed analyses, we propose two additional categories of the ECDC classification: first, “Confirmed present” with multiple observations over multiple years at the same location; second, “Follow-up absent,” where a species has been introduced but follow-up surveys yielded negative results. This will make monitoring efforts clearer and the results more detailed while still allowing for international comparisons as well as comparisons with other vectors.

While *Ph. neglectus* is the primary vector of *Leishmania infantum* and a potential vector of phleboviruses in the Balkans, and *Ph. papatasi* can transmit both *Leishmania major* and SFSV [82, 83], the vector capacity of *Ph. mascitti* has not yet been confirmed experimentally, although this species is a suspected vector for *L. infantum*. With one exception [29], neither *Leishmania* DNA nor phlebovirus RNA has yet been detected in sand flies in Central Europe. However, rising numbers of imported cases of leishmaniasis in dogs and humans have been reported, and the increasing import of pets, especially dogs, from areas endemic for *Leishmania* and phleboviruses may lead to the emergence of local transmission cycles when overlapping with local sand fly populations [84]. In addition, there have been suspected cases of autochthonous transmission of *L. infantum* in Germany and Austria, some close to known sand fly populations [13, 85, 86]. However, no autochthonous transmission by sand flies has been proven to date, and the same applies to phleboviruses. A rigorous assessment of vectorial competence of *Ph. mascittii* is undermined by the lack of available laboratory colonies of this species [87]. This may be circumvented by experimental set-ups using field-collected specimens from populations thoroughly screened as *Leishmania*-negative.

Although no *Leishmania* DNA was detected in our study, several other genera of Trypanosomatidae were found in sand flies and rodents. However, the trypanosomatid species found in the sand flies were others than those detected in screened rodents at the same trapping sites. The detection of *Trypanosoma* DNA in sand flies may be the result of accidental infections; however, other studies have also reported the detection of *Trypanosoma* spp. in sand flies. While in *Sergentomyia* primarily anuran trypanosomes have been detected [88, 89], Calzolari et al. [90] isolated *T. theileri* from *Ph. perfiliewi* with unclear vector importance. Although low interaction between local sand flies and rodents can be assumed, they deserve attention, as experimental infection of rats with *L. infantum* by *Ph. perniciosus* has been shown under laboratory conditions [91]. In addition, *L. infantum* transmission cycles between *Ph. perniciosus* and rats, for example, have been reported from urban sewage systems in Madrid and in wild biotopes and thus deserve attention [91–94].

Positivity of *Ph. mascittii* to *Trypanosoma* phylogenetically close to *T. corvi* and *T. culicavium* suggests blood feeding on birds, either domestic or wild. However, repeated positivity for *Trypanosoma theileri* complex suggests feeding on bovines (cattle, sheep, goat) and/or Cervidae (deer). These findings suggest that *Ph. mascittii* is an opportunistic species feeding on both birds and mammals. This is in line with previous findings showing that sand flies have a wide host spectrum, varying between species and including domestic animals and livestock such as cats, dogs, chickens, horses, and cattle, but also wild animals such as deer and even birds [22, 73, 95]. This flexibility can vary at a microhabitat scale, thereby influencing transmission of SFBPs depending on the available host species [96, 97]. Although domesticated animals, especially those imported from endemic countries, pose a more immediate risk for the transmission to humans, wild animals can act as important reservoirs for SFBPs, since they can act as a bridge from sylvatic to (peri-)urban transmission cycles, boosted by multi-host feeding sand fly species [98, 99].

In various studies, including ours, activity of sand flies in Central Europe has been observed from June to September with peak activity in July [16, 22]. Contrarily, Mediterranean countries such as Spain and Italy regularly report longer activity periods with bimodal activity generally peaking in July and September, with occasionally even up to three peaks (e.g. Cyprus) [75, 100, 101]. Clearly, mean annual temperatures and average temperature during the warmest month influence sand fly activity, but their reproduction also depends on the length of the warm season, and both parameters have been affected by massive changes in Central Europe in recent years [102]. In Italy, a northward spread of sand flies has been documented, and larger regions in Central Europe were modeled to become increasingly suitable for sand flies in the future [27, 103, 104]. Therefore, ecological habitat requirements should be studied in more detail.

In our study, we observed a predominance of urban sand fly locations, which may be attributed to a sampling bias, as urban habitats close to humans are regularly sampled, while more rural areas have been neglected [16, 21]. However, linear regression revealed forests as important habitats, which is supported by recent findings from Hungary, where *Ph. neglectus* and *Ph. mascittii* have been trapped at quarries and rock formations, highlighting the role of rural habitats as stepping stones in fragmented anthropogenic landscapes [73]. Therefore, future surveys should include more rural or even sylvatic sites to better understand current sand fly distribution and to assess potential emergence of new species in Central Europe.

## Conclusions

Our integrative study aimed to fill current knowledge gaps on sand flies in Central European countries, thereby updating their distribution and ecological preferences. A northward spread of sand flies due to climate change seems likely and deserves monitoring. Particularly, the lack of a natural barrier between Austria and Hungary, which is endemic to four sand fly species, might facilitate spreading of confirmed vector species in the future, and environmental changes promote the emergence of zoonotic transmission cycles involving both humans and animals in Central Europe, making this a One Health challenge.

Highly necessary surveillance should integrate entomological surveys, collection of microclimatic data, data on land-use patterns, and genetic analyses to assess population connectivity and emergence of new species.

## Supplementary Information

Additional file 1. Figure S1. The results of elbow method (a) and silhouette scoring (b) related to the optimal number of ecological clusters (*k*).

Additional file 2. Figure S2. Ecological clusters of sand flies determined by k-means clustering projected to a biogeographical map of Europe. Factors on the biplot vectors: eros = precipitation erosivity, bio12 = annual precipitation, bio13 = precipitation of wettest month, bio16 = precipitation of wettest quarter, bio18 = precipitation of warmest quarter.

Additional file 3. Table S1. Information on sand fly trapping sites.

Additional file 4. Table S2. Estimates, standard errors, z values, and *P*-values for the generalized linear model based on combined CLC categories.

Additional file 5. Table S3. Estimates, standard errors, z values, and *P*-values for the generalized linear model based on combined population categories.

Additional file 6. Table S4. COI barcoding sequences of *Phlebotomus mascittii* stored in GenBank, including voucher, location, and trapping date.

## Data Availability

Data are provided within the manuscript or supplementary information files. Trypanosomatidae sequences created in this study are available in GenBank under accession nos. PV560246–PV560260. COI barcoding sequences, vouchers, location, and date of trapping of Austrian and German **Phlebotomus mascittii** specimens are available in Additional file 6: Table S4.

## Abbreviations

CL: Cutaneous leishmaniasis
CLC: CORINE land cover
SFBDs: Sand fly-borne diseases
SDBPs: Sand fly-borne pathogens
SFSV: Sandfly fever Sicilian virus, *Phlebovirus siciliaense*
TOSV: Toscana phlebovirus, *Phlebovirus toscanaense*
VL: Visceral leishmaniasis
