# Supplementary figures and images for: The knowns and unknowns of phlebotomine sand flies (Diptera: Psychodidae) in selected countries of Central Europe
Source: Parasit Vectors. 2025 Nov 29;19:6. doi: 10.1186/s13071-025-07160-9 (PMC12771879; doi:10.1186/s13071-025-07160-9)

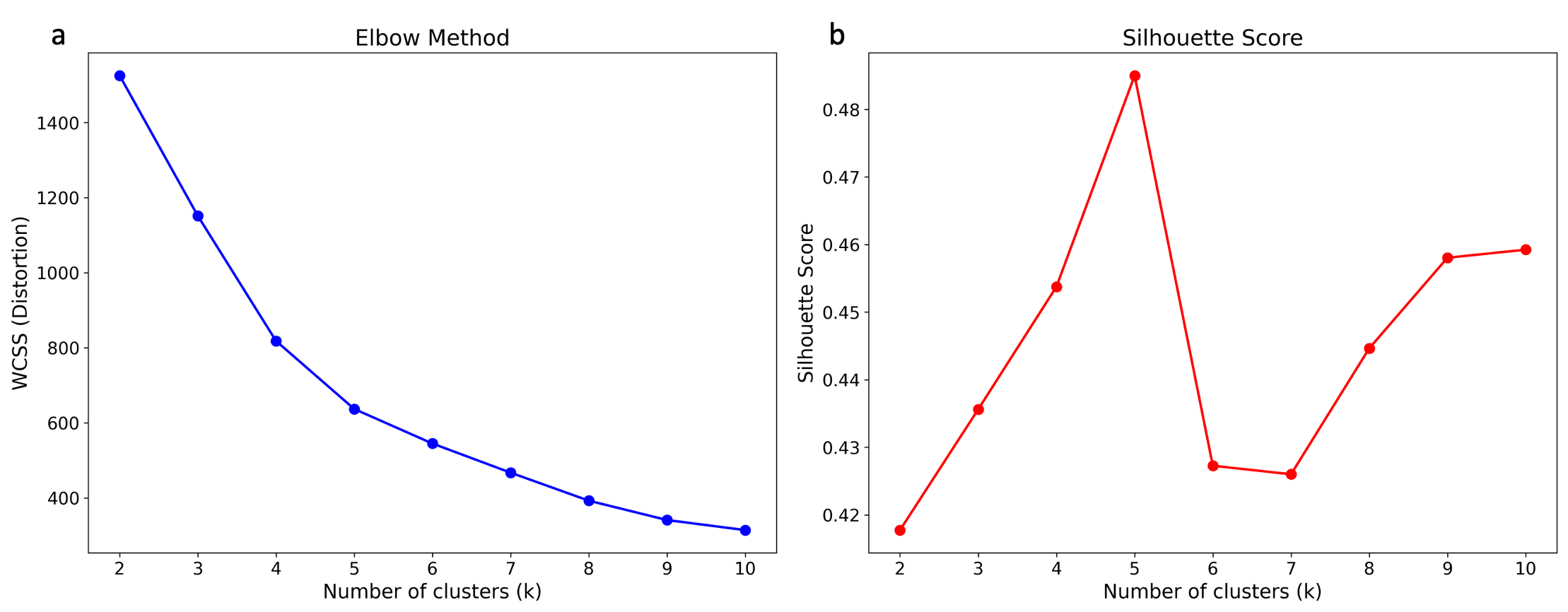

Supplement: Supplementary file 1 — Additional file 1. Figure S1. The results of elbow method (a) and silhouette scoring (b) related to the optimal number of ecological clusters (k). [file 13071_2025_7160_MOESM1_ESM.png]

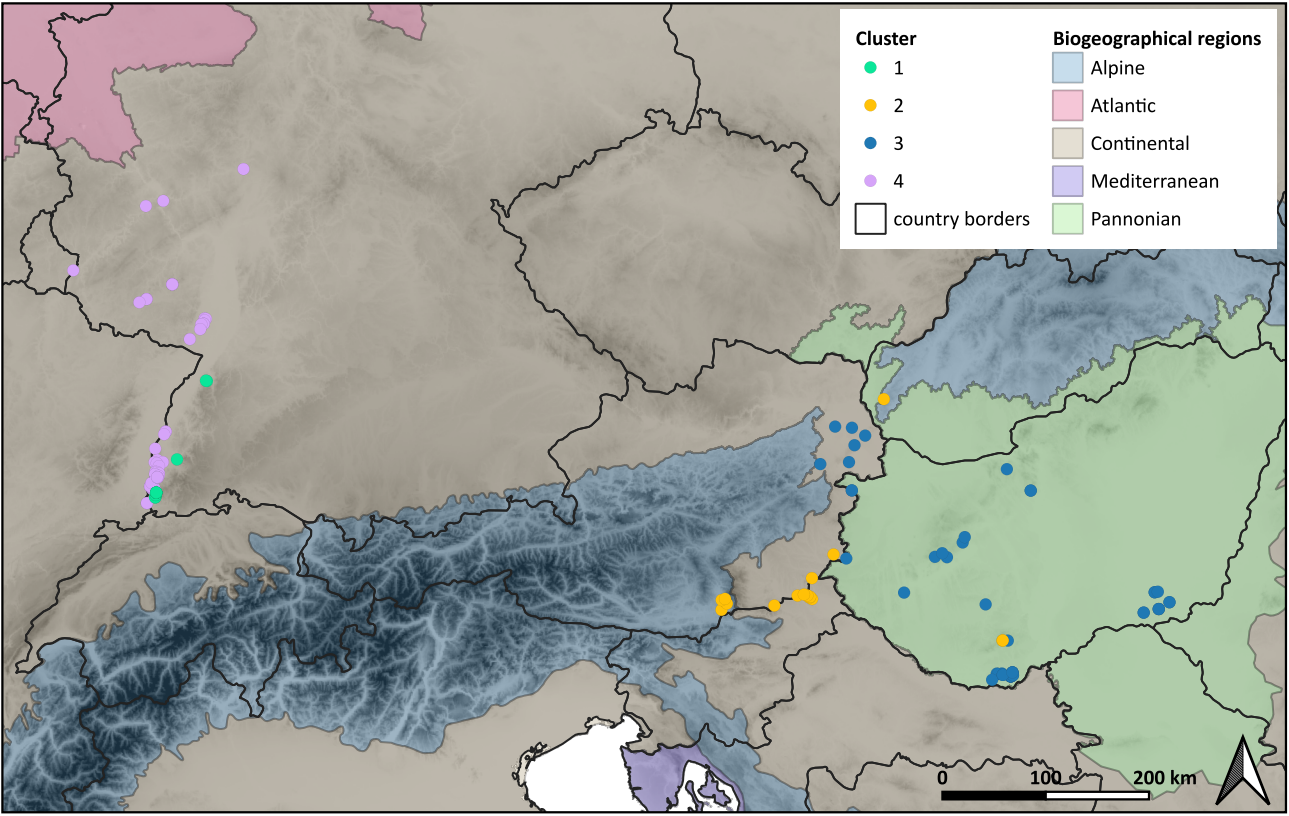

Supplement: Supplementary file 2 — Additional file 2. Figure S2. Ecological clusters of sand flies determined by k-means clustering projected to a biogeographical map of Europe. Factors on the biplot vectors: eros = precipitation erosivity, bio12 = annual precipitation, bio13 = precipitation of wettest month, bio16 = precipitation of wettest quarter, bio18 = precipitation of warmest quarter. [file 13071_2025_7160_MOESM2_ESM.pdf]
