# Supplementary material for: The knowns and unknowns of phlebotomine sand flies (Diptera: Psychodidae) in selected countries of Central Europe
Source: Parasit Vectors. 2025 Nov 29;19:6. doi: 10.1186/s13071-025-07160-9 (PMC12771879; doi:10.1186/s13071-025-07160-9)
Supplement: Supplementary file 4 — Additional file 4. Table S2. Estimates, standard errors, z values, and P-values for the generalized linear model based on combined CLC categories. [file 13071_2025_7160_MOESM4_ESM.docx]

**Supplementary Table 2.** Estimates, standard errors, z values and *P*-values for the Generalized Linear Model based on combined CLC categories.

| **Coefficients** | **Estimate** | **Std. Error** | **z value** | ***P*-value** |
| --- | --- | --- | --- | --- |
| (Intercept) | -2.2850 | 0.2189 | -10.440 | <0.001 |
| CLC_combinedForest | 1.1324 | 0.5169 | 2.191 | 0.03 |
| CLC_combinedSemi-natural Vegetation | 0.7446 | 0.5003 | 1.488 | 0.14 |
| CLC_combinedUrban | 1.0994 | 0.2505 | 4.389 | <0.001 |
| CLC_combinedWetlands | -11.2810 | 535.4112 | -0.021 | 0.99 |
| Null deviance: 644.90 on 684 degrees of freedom, Residual deviance: 621.79 on 680 degrees of freedom, AIC: 631.79 | | | | |
