# Supplementary material for: The knowns and unknowns of phlebotomine sand flies (Diptera: Psychodidae) in selected countries of Central Europe
Source: Parasit Vectors. 2025 Nov 29;19:6. doi: 10.1186/s13071-025-07160-9 (PMC12771879; doi:10.1186/s13071-025-07160-9)
Supplement: Supplementary file 5 — Additional file 5. Table S3. Estimates, standard errors, z values, and P-values for the generalized linear model based on combined population categories. [file 13071_2025_7160_MOESM5_ESM.docx]

**Supplementary Table 3.** Estimates, standard errors, z values and *P*-values for the Generalized Linear Model based on combined population categories.

| **Coefficients** | **Estimate** | **Std. Error** | **z value** | ***P-value*** |
| --- | --- | --- | --- | --- |
| (Intercept) | -1.362 | 0.245 | -5.57 | <0.001 |
| SubclassRural | -0.206 | 0.270 | -0.76 | 0.445 |
| SubclassUrban | 0.074 | 0.468 | 0.16 | 0.874 |
| Null deviance: 644.90 on 684 degrees of freedom Residual deviance: 643.99 on 682 degrees of freedom AIC: 649.99. | | | | |
